# Designing Supportive e-Interventions for Partners of Men With Prostate Cancer Using Female Partners’ Experiences: Qualitative Exploration Study

**DOI:** 10.2196/31218

**Published:** 2022-02-15

**Authors:** Natalie Winter, Anna Green, Hannah Jongebloed, Nicholas Ralph, Suzanne Chambers, Patricia Livingston

**Affiliations:** 1 Centre for Quality and Patient Safety Research Institute for Health Transformation Deakin University Geelong Australia; 2 School of Nursing Deakin University Geelong Australia; 3 Faculty of Health University of Technology Sydney Australia; 4 Centre for Health Research University of Southern Queensland Toowoomba Australia; 5 School of Nursing and Midwifery University of Southern Queensland Toowoomba Australia; 6 Faculty of Health Sciences Australian Catholic University Brisbane Australia; 7 Faculty of Health Deakin University Geelong Australia

**Keywords:** prostate cancer, prostatic neoplasms, e-intervention, smartphone, qualitative research, caregivers, mHealth, mobile phone

## Abstract

**Background:**

Partners of men living with prostate cancer (PCa) can experience a variety of unmet needs that are largely unaddressed by health care professionals. There is limited evidence to suggest which approach may be most effective in supporting partners’ unmet needs and further research is required to determine how to provide support to caregivers and how technology solutions can be designed.

**Objective:**

This study aims to explore the experience of partners of men living with PCa and their perceptions of the potential role of information technology in supporting their needs.

**Methods:**

A qualitative descriptive methodology using focus groups and phone interviews was used. Purposive sampling was used to recruit people attending a national conference supported by a national PCa organization. Interview guides were adapted from an existing evidence-based smartphone app for caregivers of people with colorectal cancer. Sessions were audio recorded and transcribed verbatim. A coding framework was developed, and transcripts were coded line by line into the framework. Codes within the framework were grouped into descriptive categories that were then developed into analytical themes.

**Results:**

A total of 17 female partners participated in the study, with an average age of 64 (SD 8.5) years. The following two main themes emerged: In the first theme, that is, *How technology can be shaped to support female partners of prostate cancer survivors*, the content and design of the smartphone app was discussed in addressing female partners’ needs. The following four subthemes were developed: getting support from social networks and resources, the lack of relevant information, demystifying future care expectations during and following a PCa diagnosis, and delivering the smartphone app—to whom and from whom. In the second theme, that is, *The benefits and barriers of technology,* the suitability of smartphone apps as a supportive modality for female partners was described. This included three subthemes: the smartphone app as an appropriate modality for supporting female partners, the future anticipated benefits of using the smartphone app, and concerns for storing and accessing information on the internet.

**Conclusions:**

A smartphone app may be a suitable modality for providing information and peer support to female partners of men living with PCa. There is a need to provide peer support for female partners in future interventions to ensure that female partners’ intimacy and daily practical needs are met.

## Introduction

### Background

Globally, prostate cancer (PCa) is the most commonly occurring cancer in men [1], with over 16,000 men diagnosed in Australia in 2020, accounting for approximately 20% of all male cancer diagnoses [2]. PCa survivorship research has predominantly focused on the psychological and physical effect of PCa treatments on men [3]. The psychosocial impact on their partners is an emerging area of priority, with research suggesting they may experience greater levels of distress than the individual with PCa [4-6], which may be attributed to avoidant communication between patients and partners [7]. Partners also report their own needs often go unaddressed by health care professionals [8]. As evidence of acceptable and effective interventions to support the partners of men with PCa remains unclear [9], further understanding of partner- or caregiver-specific issues is increasingly recognized as important to inform evidence-based supportive interventions [3,9]. A clearer understanding is needed to determine whether e-interventions can be adapted to meet partners’ specific needs.

Dyads refer to the patient and spousal partner, and dyadic interventions remain an area of uncertainty within PCa. For instance, interventions are patient focused and produce conflicting results between patient and partner outcomes [9]. Unmet needs refer to areas where support may be lacking [10] and often can be organized several categories, including access to services, psychological care, financial support, relationships and communications, information, and spirituality [11]. Caregivers of people with cancer in general experience unmet needs related to providing symptoms and side effects management and maintaining function and caring for themselves [12]. Caregivers often experience elevated levels of distress across the disease trajectory [13]—less than 40% of caregivers participate in social events [14] and many experience financial burdens associated with loss of employment related to changing health status of the cancer survivor or increased caregiving responsibilities at home [15]. Previous studies have identified that there are over 200 unmet needs or issues that caregivers may experience [16]. Although there is the requirement to deliver interventions for the most distressing needs of caregivers, it is also imperative to provide caregivers with access to support and resources to address their less common needs [17]. Although flexibility in technology designs provide the potential to meet a range of unmet needs experienced by partners of men with PCa by having the capacity to tailor programs to users’ needs, how best to design and deliver e-interventions requires more investigation.

e-Interventions and smartphone apps in particular have the potential to deliver resources to large groups of people [18] and facilitate the delivery of individually tailored care. The majority of Australians currently own smartphones and use of smartphones app is expected to increase [19]. Smartphone apps offer flexibility when seeking information and support as they allow caregivers to locate resources privately and from anywhere in the world with an internet connection [20]. e-Interventions have previously been used among caregivers of people with cancer and provide promising results; however, there is limited information about the use of smartphone apps [21]. A previous pilot study of a smartphone app for caregivers of people with colorectal cancer found that smartphone apps can be useful for caregivers when managing their own needs and that resources such as this should be available to all caregivers in a similar situation [22]. To be beneficial, smartphone apps should be highly relevant and appropriate to the needs of caregivers and easy to access [22,23].

A smartphone app has been developed using a user-centered design approach for caregivers of people with cancer [24] and trialed among caregivers of people with colorectal cancer [22]. This smartphone app was found to be feasible for caregivers and acceptable, with 85% of caregivers stating that they think the smartphone app should be made available to other caregivers looking after another adult with cancer [22]. This smartphone app, called Carer Guide, addressed unmet needs that caregivers commonly experience such as cancer information, including diagnosis, treatment, side effects, and symptoms; their mental well-being; lifestyle tips for caregivers, including diet and exercise; financial allowances and legal tips; and hospital contacts and information. Currently, no smartphone apps exist to support caregivers looking after men with PCa and the needs of these caregivers in relation to a technology solution is unclear. Therefore, this study aims to explore the experiences of partners of survivors of PCa as caregivers and their perceptions of the potential role of a smartphone app in supporting their unmet needs across the stages of PCa diagnosis.

### Objectives

Using a qualitative study design, the objectives of this study are as follows:

1. Explore the experiences of partners of survivors of PCa as caregivers and how a smartphone app may support their unmet needs.
2. Identify partner preferences around the potential role of smartphone app in supporting their needs.
3. Obtain feedback on an example smartphone app and the potential role of a generic platform that can incorporate different aspects of PCa disease progression during the life course of survivors of PCa.

## Methods

### Setting

This study included two parts: (1) one focus group at an Australian national conference day held in Brisbane, Queensland, for PCa and (2) phone interviews with partners of men with PCa recruited nationally from an Australian PCa organization registry. The first part was conducted in July 2019, and recruitment for the second part occurred between May and June 2020. This study received approval from the Human Research Ethics Committee of the University of Technology Sydney (ETH19-3700) and Deakin University (2019-244).

### Participants

Purposive sampling was used to recruit eligible partners.

#### Inclusion Criteria

Inclusion criteria included eligible partners (male or female) of men diagnosed with PCa at any stage of the disease and aged ≥18 years.

#### Exclusion Criteria

Exclusion criteria included people who were unable to follow conversations in English language.

In the first part, recruitment flyers advertising the date and location of the focus group were disseminated through the networks and caregiver support groups of the Australian PCa organization. In the second part, a recruitment flyer was released within the Australian PCa organization registry of people willing to be involved in research. Partners interested in participating in the project initiated contact with the research team (NW or AG) by phone or via email. Interested partners were emailed a copy of the plain language and consent form that was either signed on the day of the conference or returned via email before phone interviews.

### Data Collection

This study was conducted using a qualitative descriptive design [25], informed by the epistemology of pragmatism that seeks to ascertain whether the knowledge generated has served the specific purpose of the study [26]. The focus group was facilitated by NW, AG, and PML and was attended by one nurse counselor as an observer to the group. The nurse counselor was an attendant at the conference and was external to the research project. The focus group was held in a quiet room away from the main conference. The phone interviews were conducted by NW. The focus group and phone interviews were guided by the same semistructured questions with prompts. The inclusion of phone interviews allowed us to invite people living across Australia and allowed the research to continue during COVID-19 pandemic lockdowns and when partners were unable to attend focus group sessions. The same questions and prompts were used in both focus groups and phone interviews to ensure consistency across sessions. The questions had been used in a previous study aimed at developing a smartphone app for caregivers of people with cancer [24,27].

**Figure 1:**
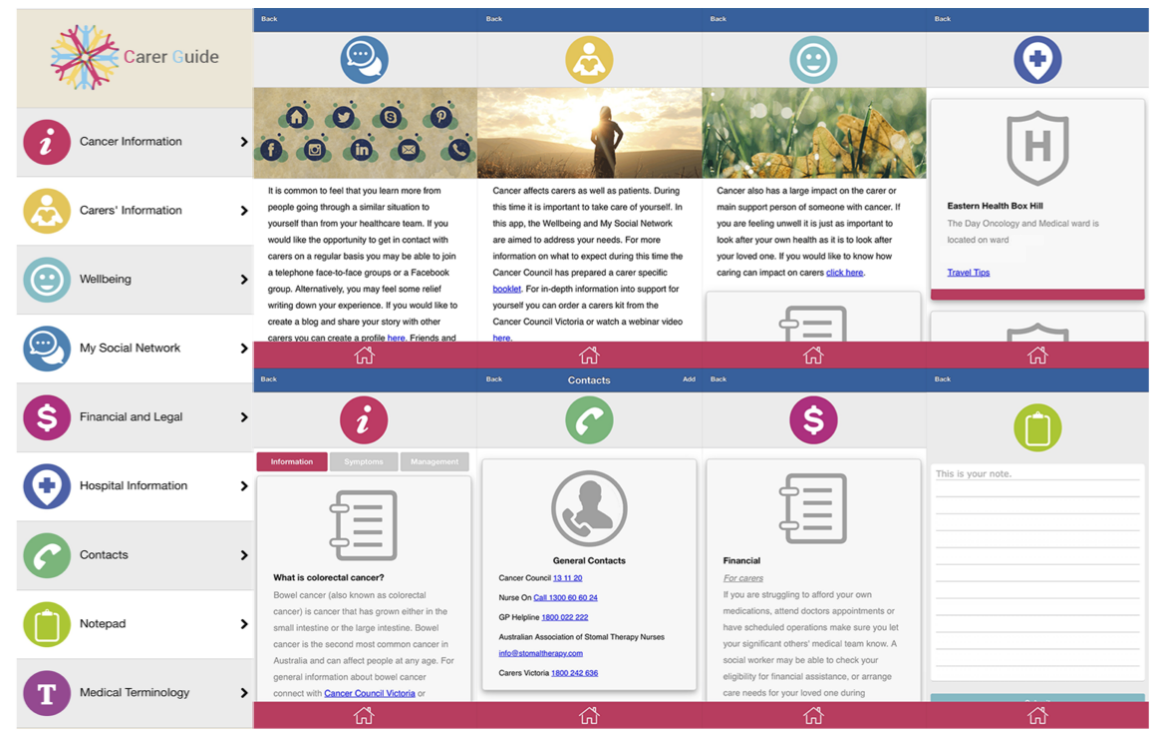
Previous smartphone app (Carer Guide) with content displayed for partners to review: Cancer Information, Carer Information, Wellbeing, My Social Network, Financial and Legal, Hospital Information, Contacts, Notepad, and Medical Terminology.

Partners were asked questions about their experiences with PCa, how they found support, and the suitability of smartphone apps to meet their unmet needs. To support discussion, screenshots of an existing smartphone app for caregivers of people with colorectal cancer [24] (Figure 1) were shown to partners who were then asked to respond to questions about how the smartphone app may be specifically adapted to meet the needs of partners of men with PCa. The original smartphone app *Carer Guide* was developed using a user-centered design approach [28] and provided a source of information and resources specific to caregivers identified unmet needs. Primarily *Carer Guide* was a static source of information with supporting resources, including notepad and contacts for caregivers to enter their information as required. The potential to expand the smartphone app to include tailored features to engage with users is the aim of this study. The focus group and phone interviews were audio recorded and transcribed verbatim. Data collection continued until data saturation occurred.

Demographic data were collected from partners who participated in phone interviews. Partners who attended the focus group were invited to complete a demographic questionnaire; however, this was not mandatory. Completed data were collected for 11 partners.

### Data Analysis

#### Overview

Transcripts were read twice by 2 researchers (NW and AG) and a framework analysis was used based on initial impressions of overarching concepts [29]. Two authors (NW and AG) independently developed key codes; the authors then discussed the codes and agreed upon the codes that would be included in the framework. Transcripts were then coded line by line using NVivo software (QSR International) into the coding framework by one author (NW) and were checked by a second author (AG). Codes were grouped into similar and contrasting descriptive categories, which were then developed into themes and subthemes with interpretation confirmed by the full authorship team. Concepts were similar between focus group and phone interview transcripts, and thematic analyses were subsequently combined. Demographic data were analyzed using descriptive statistics. This study followed the COREQ (Consolidating Criteria for Reporting Qualitative Research) guidelines for qualitative studies [30].

#### Rigor

To ensure trustworthiness of the findings the following steps were taken as recommended by Bradshaw et al [31]. Credibility was promoted by establishing a trusting relationship and rapport with partners during the consenting process before conducting focus groups and phone interviews, and empathy was provided during sessions [31]. To provide confirmation of results, field notes were taken during focus groups and phone interviews to confirm major and minor themes from the thematic analysis process [31]. Demographic information was collected from partners where possible, and direct quotes were used to demonstrate findings [31]. An audit trail was used to provide dependable study procedures and results. To enhance the transferability purposive sampling was used [31].

#### Research Team

The research team consisted of psycho-oncology researchers with backgrounds in nursing, psychology, and social science who cumulatively had over 50 years of experience in providing support to people with cancer.

## Results

### Demographic Characteristics

A total of 17 female partners participated in the study—8 (47%) in the focus group and 9 (53%) in phone interviews. The average age of female partners was 64 (SD 8.5) years, and all were living with their male partners diagnosed with PCa. Complete demographic data are given in Table 1. Of the 9 partners in phone interviews, 8 (89%) provided information about the state they resided in Australia: 62% (5/8) lived in New South Wales, 12% (1/8) in Victoria, 12% (1/8) in Queensland, and 12% (1/8) in Western Australia. The focus group ran for 112 minutes, and phone interviews were on average for 47 (SD 12; range 31-67) minutes.

**Table 1:** Demographic data of female partners (n=11).

Characteristics		Value
Age (years), mean (SD)		64 (8.5)
Female, n (%)		11 (100)
Residing with patient, n (%)		11 (100)
**Highest level of education, n (%)**		
	Secondary school	2 (18)
	Certificate or diploma	4 (36)
	University degree	4 (36)
	Postgraduate degree	1 (9)
**Current or past caregiver, n (%)**		
	Current	8 (72)
	Past	3 (27)
**Length of time in the caregiver role,** **n (%)**		
	6 months to 1 year	1 (9)
	1 year to 2 years	1 (9)
	>2 years	9 (81)
**Treatment received by the partner,** **n (%)**		
	Hormone therapy	5 (45)
	Not currently receiving treatment	5 (45)
	Drug trial	1 (9)
**Self-identification as a caregiver,** **n (%)**		
	Yes	6 (54)
	No	5 (45)

### Findings

#### Overview

In addition to concepts already outlined in the *Carer Guide* app (Figure 1), female partners suggested content and resources that could support partners across the PCa survivorship continuum. Two overarching themes emerged from the data: *how technology can be shaped to support female partners of PCa survivors* and *the pros and cons of technology*. An overview of the themes and subthemes is given in Textbox 1.

**Themes and subthemes derived from the thematic analysis.**

**How technology can be shaped to support female partners of survivors of prostate cancer**

- Support from social networks and resources
- The lack of relevant information
- Demystifying future care expectations during and following a prostate cancer diagnosis
- Delivering the smartphone app—to whom and from whom

**The benefits of and barriers to technology**

- A smartphone app as an appropriate modality for supporting female partners
- The future anticipated benefits from using the smartphone app
- Concerns for storing and accessing information on the internet

#### How Technology Can Be Shaped to Support Female Partners of PCa Survivors

In this theme, female partners discussed how smartphone apps could be designed to meet their needs. Four subthemes emerged highlighting the unmet needs of female partners and included support from social networks and resources; the lack of relevant information; demystifying future care expectations during and following a PCa diagnosis; and delivery the smartphone app—to whom and from whom.

#### Support From Social Networks and Resources

Informal social networks of friends and family members, as well as more formal networks of partner support groups, were the main source of support for female partners of men with PCa. Benefits from attending support groups included shared learning, emotional support for intimate and communication issues within the dyad as a result of the PCa diagnosis, and support for female partners’ own individual needs:

They [women] tell you exactly what’s happening to their husbands and their impotence and their sexual life. And women are very honest.

ID3

The inclusion of support networks within a smartphone app was, therefore, a key feature to include, for female partners in particular, to streamline and improve access to this source of support, which was, at times, difficult to find independently:

Initially finding information about support groups and things like that, that was quite hard to find.

ID1

In recognition of diverse preferences for sharing information among female partners, one woman also suggested the inclusion of videos of couples discussing their experiences of PCa may be useful for partners who preferred not to access information and support from support groups:

Hearing those two [information video of a couple affected by prostate cancer] talk about...their sex lives and how that had changed for them...I actually found that really useful actually hearing people talk about their experience...And you know, if you, if you didn’t want to go off to a support group, or if you didn’t have a support group available to you, just being able to sit down and even replay those interviews or absorb them at your own pace, I think was really, was really worthwhile.

ID9

Similarly, a gap in knowledge of available resources was evident. Female partners reported their ack of knowledge on what they entitled to, including access to Prostate Cancer Nurses and community resources, and identified that this could be addressed in an app by highlighting available services to female partners:

Where do you find out about how you can get some funded counseling? Where do you go?

ID4

#### The Lack of Relevant Information

Female partners described information related to the diagnosis of PCa, treatment, and treatment outcomes was lacking. Often, side effects of treatment were understated and the lasting side effects of hormonal therapy were poorly explained by clinicians and information sources:

There might be sexual dysfunction...You know it’s like just a little dot point on the page when...I feel like that should really be highlighted more for partners.

ID9

Some female partners expressed frustration at the representation of PCa as a *safe cancer*, as this was not their experience, and the lack of available information was insufficient to meet their understanding:

I would have liked to have understood better just how big a risk he was at...I don’t think that that’s made very clear...You know Gleason 9 is like exponentially worse than Gleason 7...I don’t think I appreciated how much at risk he was for recurrence.

ID4

Female partners also noted that information should be specific for couples at different stages in their life, such as those still wanting to have children later in life:

Some partners may, particularly if they’re younger...there would have to be a whole message about you need to go and...get sperm frozen.

ID9

Having had access to a smartphone app to find PCa-specific information was noted as a valuable resource that could have saved time, improved access to information, and reduced anxieties of female partners:

I think it [having an app] would just expedite the information gathering. Having a one stop shop.

ID3

I would still have had the anxiety about how successful his treatment is going to be. But if I had a bit more understanding of what was going...[it] would have taken a bit of the edge off in terms of the anxiety.

ID2

#### Demystifying Future Care Expectations During and Following a PCa Diagnosis

Female partners identified the need for clear information on what to expect and what care they would need to provide following their partners’ diagnosis. This often included practical tips that were learned through informal networks or from experience:

I found a couple of people that I’ve spoken to, is where to get supplies from, like incontinence pads and how they cope with catheters when they get home.

FG

We just sort of did a lot by ear...I looked at my husband when he was in hospital and thought you won’t be able coming out in jeans. I need to go and get tracksuit pants and ran off to the shops and bought tracksuit pants.

ID1

Female partners also felt that the app may provide them with information about what partners can expect at each stage of the cancer journey:

He’ll have his own journey, he’ll be doing his own thing, but this [information provided] is what you...can expect over the next you know, 3 months, 6 months, 12 months, 2 years, 3 years.

ID9

[Including] a what to expect kind of tabs...‘cause at the outset that’s really important.

ID2

In particular, some female partners noted wanting to feel prepared for end-of-life care and what would happen when the health of their partner deteriorated or how to manage future funeral arrangements:

Can I cremate him? You know, it took me three weeks to find an answer. No, you can’t. You can’t cremate him straight away. You’ve got to wait; I think it was 20 days or something like that...because of the radiation [from brachytherapy].

FG

#### Delivering the Smartphone App: To Whom and From Whom

There was variability around who female partners felt the main audience of the smartphone app should be. Some female partners felt that the smartphone app should be tailored specifically to partners:

I think it might be good to have an app just for carers actually, because then you’re focusing on them...not the person with cancer and sometimes it is the carers who, who needs the help.

ID7

Alternatively, some female partners reported that the smartphone app should be delivered to both partners and patients or that both people in the relationship should have access to all of the information:

Maybe it will be better to have it for carers and patients together. Because then I’m a nosey person and I want to know what’s going on.

ID1

The name of the smartphone app had an impact on female partners’ perception of whether the smartphone app was meant for them. Some female partners responded positively to the term *caregiver*, whereas others felt that this did not match their experience:

I would think oh that’s probably going to apply to people you know who are much older than me. That’s what I think of when I hear the word carer.

ID9

Female partners agreed that the smartphone app should be supported or recommended by reputable sources to encourage them to download it, such as health care professionals, cancer organizations, or advertisements within hospitals:

If it was sitting there in the waiting room [outpatient oncology], I would have picked it up and I, I would have looked at it. I would have logged on and checked it out.

ID2

#### Benefits of and Barriers to Technology

In this theme, the suitability of apps as a modality to support the female partners of men with PCa was explored. The three subthemes that emerged around are the smartphone app as an appropriate modality for supporting female partners, the future anticipated benefits from using the app, and concerns around storing and accessing information via internet.

#### A Smartphone App as an Appropriate Modality for Supporting Female Partners

Female partners responded positively to the layout of the *Carer Guide smartphone* app because the app was easy to navigate and its design was suitable for people looking after someone with cancer:

It’s colorful, it’s bright, it’s cheery.

ID3

I like the menu, it looks really easy to navigate.

ID9

The purpose of the app was clear, and the original content covered aspects of care that female partners felt were important in supporting caregivers of men living with PCa:

I think that [a smartphone app] would be a help for a lot of people especially when their partners are newly diagnosed it’s very difficult to understand it all. If there was one [smartphone app] just for carers that would give them some information that’s specifically to them...it would have been helpful...It [the Carer Guide] goes through quite a few things that I think are interesting yeah, I think that you need to know.

ID7

Many female partners stated that they kept medical records and contact details separately, either in paper files or within regular mobile phone functions. General consensus among female partners was that storing this information within the smartphone app would be beneficial for quickly and easily locating information during emergencies, creating digital notes rather than numerous paper documents, and compiling all relevant information at the same location:

I think it’s great that you can have contact numbers in there. Um, I struggle sometimes with finding, you know, who do you ring...especially if it’s at 11 o’clock at night, and there’s lots of pain.

FG

I can see how this [the app] could be useful and give you some way to put everything that it wasn’t um it wasn’t just all over the place...one of the things that everybody tells you as soon as you get diagnosed is start a binder with all your results...in some ways this [the app] could potentially complement that as a digital binder.

ID4

The use of reminders was predominantly discussed for daily care tasks such as medication and appointment reminders. One woman described that reminders and bookmark functions could also be used for her own needs when allocating time to visit certain sections of the app:

You could kind of do a follow up with the app...set yourself a reminder that something pops up somewhere in your calendar to say hey, don’t forget to read this section you know you bookmarked this for yourself.

ID9

Another female partner expressed that storing this information within the *Carer Guide* smartphone app would be preferable and beneficial for her mental well-being compared with other smartphone apps available on her phone:

We put all that stuff [scheduling] in our outlook calendar, so then it pings at you and reminds you when you don’t really want to know about it...That would be really nice to have in its own little world when you’re in the right mind frame to go and look at it...Even as someone who’s not actively dealing with those sort of treatment schedules...when we [are waiting to] get the results back...that’s when I’m most on edge...so putting that appointment reminder somewhere else [in the Carer Guide app] would be nice.

ID4

Two female partners identified that they would be unlikely to use a smartphone app as a modality for support. Other female partners suggested that although they use smartphone apps, levels of technology literacy and comfort in using smartphone apps may vary for other female partners:

Well see I probably wouldn’t use it very much.

FG

But I’ve never actually gone into the app store and just put in the word prostate to see if there’s a – there may be something there. I – I don’t know. That’s not how I search for stuff.

P1

Well, it’s because we’re not used to doing that, these old people...these apps are very new.

P2

#### The Future Anticipated Benefits From Using the Smartphone App

Reflecting on how access to the *Carer Guide* smartphone app may have supported them in earlier stages of their caregiver journeys, female partners identified a number of potential benefits. Benefits included having the app as a *one-stop shop* for everything that they needed while *on the run*. In the context of providing care to their partners with PCa, female partners identified that the smartphone app would have been a useful source of information to understand more about what was happening and what they needed to learn more about:

It would have given me a good structure to start knowing what I needed to get organized and potentially what I needed to start asking questions on.

ID4

Female partners also felt that access to the smartphone app would have provided them with the opportunity to seek information to address their own needs without the patient knowing about it:

[in reference to whether the app would help in finding information for caregivers] Definitely, definitely. ‘cause sometimes I would actually feel a bit guilty for looking up stuff for myself.

ID2

#### Concerns for Storing and Accessing Information via the Internet

Some areas of concern when using technology included the security of personal information stored on the internet and hesitation toward possibilities of accessing overwhelming information that can lead to distress. The ability to input medical information or link the smartphone app with medical record platforms such as MyGov was noted as benefits of the smartphone app for storing up-to-date medical information. However, concerns over risk of web-based information breach was noted among female partners:

What about keeping a medical record?...The app would have to be very secure.

FG

Similarly, female partners noted that the inability to understand overwhelming medical content available on the internet along with lack of clinical support to explain the web-based content was a potential concern for some partners:

When you’re given a booklet by the doctor...it’s actually an opportunity for somebody to discuss it. Whereas when you go searching for stuff on the Internet...if you find too much in-depth information it can panic you.

ID5

## Discussion

### Principal Findings

Overall, female partners of men living with PCa responded positively to the potential use of a smartphone app as a modality of support during their cancer journey. A previous smartphone app designed for caregivers of people with colorectal cancer, *Carer Guide* [21], was acceptable to female partners, and the original content (cancer information, carer information, well-being, social network, hospital information, financial and legal, and medical terminology) was appropriate to their needs. Content specific to the shared experiences of these female partners and additional content that may be helpful to include in future e-intervention designs included more prominent linking with peers and support groups, information and support related to the effects of PCa treatment on their intimate relationships, and more clarity on what to expect during each stage of the caring period. The need for flexibility with future interventions was apparent, as some female partners preferred to have access to a smartphone app specifically for caregivers, whereas others thought it should be a resource shared between the patient–caregiver dyad. There is a need to ensure users are aware of the level of security that can be offered by digital health devices, particularly when they are being used to input or store medical information to ensure privacy and confidentiality.

Addressing the patient–partner dyad intimate relationship during a PCa diagnosis is a primary area of focus within the literature [32]. Female partners in this study again highlighted the impact of PCa on their intimate relationship and their ability to seek support for themselves, suggesting this is as an ongoing unmet need. Dyadic research has shown that partners’ needs as caregivers in the literature are not well-addressed in the PCa field [9]. Previous studies have described that strong couples’ communication can have positive effects on mental outcomes of patients and caregivers [33]. However, in this study, female partners also highlighted the need for open communication with others such as wanting practical advice from peers about how to manage frustrations with intimacy on a daily basis.

Female partners reported that practical support was also required for daily care tasks and needed incorporation into the smartphone app. There is evidence that caregivers are performing clinical tasks in the home setting [34]. However, the need for guidance on daily tasks, such as locating incontinence products, preparing for surgical recovery, and managing day-to-day needs is apparent, and there is little in the literature to suggest that these types of unmet needs are being addressed or that there are effective symptom management interventions. The need for peer support was a key approach female partners felt intimate and practical issues could be addressed. Peer support can help caregivers to find information and resources related to patient care and share emotional experience with others in a similar situation [35]. These female partners demonstrated this through the sense of belongingness and empathy when talking about intimate issues with each other. Providing a range of communication and supportive resources in the smartphone app may be more beneficial in meeting caregivers’ unmet needs related to intimate and practical issues; however, this requires further investigation.

Female partners were favorable to the concept of using a smartphone app while caring for a male partner with PCa. However, there are varied perspectives on whether resources such as Carer Guide are feasible for use for all caregivers because of personal preference for seeking information. From our previous work, it was evident that caregivers required the content and functionality of a digital resource to be specific to their situation to fulfill their unmet needs [27]. In this study, female partners requested specific information related to different stages of disease and when disease progression occurs during a PCa diagnosis. Female partners noted having information would reduce fears of the unknown and potentially reduce associated anxiety. Of note, some female partners particularly mentioned seeking information about bereavement care or care after death, suggesting that female partners would like information to feel prepared for this period of care. Unmet needs related to palliative care have previously been recorded in a systematic review and are present in 21% to 100% of participants across studies, suggesting that there is great variability in the need for information about palliative and bereavement care [36]. However, only one of these studies was within the PCa caregiver field [37], suggesting that more research is needed in this area.

An additional finding of this study was the need for flexibility in the audience of an e-intervention. PCa in particular is often seen as a couples’ disease [38]; therefore, there is a requirement to provide support for the couple going through a PCa diagnosis. However, as described, female partners can experience both unmet needs specific to their caring role and needs different from the dyadic relationship. Therefore, these individual unmet needs also require tailored support. A recent systematic review by Luo et al [39] recommended that interventions for families affected by cancer should include four components: information, communication and support, skill building, and psychoeducation. It is important, therefore, to encompass all of these domains when designing and developing interventions to enhance their potential to improve caregivers’ outcomes. Flexible designs or the ability to customize items are required to ensure apps can be tailored to caregivers’ unmet needs. Furthermore, as identified by Lambert and Girgis [17], it is necessary for e-interventions to address less common unmet needs of caregivers, highlighting the importance of flexibility in design, content, and delivery of interventions for caregivers.

In creating reliable e-interventions for caregivers, it is also paramount to consider the safety and privacy concerns of end users. As described by the female partners in this study, when entering medical or sensitive information into smartphone apps, it is vital that users are confident that they know how their information is being stored and with whom it is being shared, if anyone. Several frameworks exist to assess the different functionalities of smartphone apps. Variance in these frameworks can lead to difficulties evaluating smartphone apps [40]. To ensure the safety and privacy of end users across trials, standardized approaches for the assessment and production of smartphone apps are required. With more streamlined e-interventions, it may be possible to provide caregivers with the opportunity to engage with a greater number of web-based resources to meet their needs.

Within the clinical setting, it is of vital importance that e-interventions are supported by health care professionals during implementation into routine practice. Referrals and recommendations to existing and up-and-coming interventions are required by clinicians in outpatient and general practice settings to support partners and caregivers who regularly prioritize their own unmet needs last [41].

### Limitations

This study had several limitations, including the small sample size and homogeneity in participation of only female partners. The study was advertised for both male and female partners to participate; however, no male partners initiated contact. Furthermore, description of the study sample was limited, as not all partners who participated completed demographic questionnaires. Despite the small sample size, findings were similar to previous research describing partners’ experiences of supporting men with PCa [8]. Partners in our sample lived in several states across Australia, suggesting that experiences were similar across the country; however, this should be interpreted with caution because of the small sample size, as these findings may not be transferable to other demographic context. Future research should endeavor to assess experiences, and the adaptation of support modalities in same sex attracted men affected by PCa.

Partners were shown screenshots of the smartphone app rather than having the opportunity to use the app prototype. This may have resulted in different consequences about the usefulness of the app. However, the main intent of this study was to understand how the content could be extended to the PCa setting. In addition, 2 authors (NW and PML) were the original developers of the Carer Guide smartphone app. To reduce any potential biases in findings, multiple members of the authorship team, including those with expertise in PCa survivorship and those with no prior involvement in the original smartphone app, were involved in the adaption of the interview guide and each stage of data analysis.

### Conclusions

A smartphone app may be a suitable modality for providing information and peer support to female partners of men living with PCa. A few changes are required to adapt an existing smartphone app to partners’ specific needs. There is a strong need to provide peer support for female partners in future interventions to ensure that female partners’ intimacy and daily practical needs are met.

## Abbreviations

COREQ: Consolidating Criteria for Reporting Qualitative Research
PCa: prostate cancer

## References

[1] Bray F, Ferlay J, Soerjomataram I, Siegel RL, Torre LA, Jemal A (2018). Global cancer statistics 2018: GLOBOCAN estimates of incidence and mortality worldwide for 36 cancers in 185 countries. CA Cancer J Clin.

[2] (2021). Cancer data in Australia. Australian Institute of Health and Welfare.

[3] Hyde MK, Zajdlewicz L, Lazenby M, Dunn J, Laurie K, Lowe A, Chambers SK (2019). The validity of the distress thermometer in female partners of men with prostate cancer. Eur J Cancer Care (Engl).

[4] Cliff AM, MacDonagh RP (2000). Psychosocial morbidity in prostate cancer: II. A comparison of patients and partners. BJU Int.

[5] Eton DT, Lepore SJ, Helgeson VS (2005). Psychological distress in spouses of men treated for early-stage prostate carcinoma. Cancer.

[6] Couper J, Bloch S, Love A, Macvean M, Duchesne GM, Kissane D (2006). Psychosocial adjustment of female partners of men with prostate cancer: a review of the literature. Psychooncology.

[7] Boehmer U, Clark JA (2001). Communication about prostate cancer between men and their wives. J Fam Pract.

[8] Collaço N, Rivas C, Matheson L, Nayoan J, Wagland R, Alexis O, Gavin A, Glaser A, Watson E (2018). Prostate cancer and the impact on couples: a qualitative metasynthesis. Support Care Cancer.

[9] Chambers SK, Hyde MK, Smith DP, Hughes S, Yuill S, Egger S, O'Connell DL, Stein K, Frydenberg M, Wittert G, Dunn J (2017). New challenges in psycho-oncology research III: a systematic review of psychological interventions for prostate cancer survivors and their partners: clinical and research implications. Psychooncology.

[10] Carey M, Lambert S, Smits R, Paul C, Sanson-Fisher R, Clinton-McHarg T (2012). The unfulfilled promise: a systematic review of interventions to reduce the unmet supportive care needs of cancer patients. Support Care Cancer.

[11] Lambert SD, Harrison JD, Smith E, Bonevski B, Carey M, Lawsin C, Paul C, Girgis A (2012). The unmet needs of partners and caregivers of adults diagnosed with cancer: a systematic review. BMJ Support Palliat Care.

[12] Heckel L, Fennell KM, Reynolds J, Osborne RH, Chirgwin J, Botti M, Ashley DM, Livingston PM (2015). Unmet needs and depression among carers of people newly diagnosed with cancer. Eur J Cancer.

[13] Northouse LL, Katapodi MC, Schafenacker AM, Weiss D (2012). The impact of caregiving on the psychological well-being of family caregivers and cancer patients. Semin Oncol Nurs.

[14] Litzelman K, Reblin M, McDowell HE, DuBenske LL (2020). Trajectories of social resource use among informal lung cancer caregivers. Cancer.

[15] Bradley CJ (2019). Economic burden associated with cancer caregiving. Semin Oncol Nurs.

[16] Stenberg U, Ruland CM, Miaskowski C (2010). Review of the literature on the effects of caring for a patient with cancer. Psychooncology.

[17] Lambert SD, Girgis A (2017). Unmet supportive care needs among informal caregivers of patients with cancer: opportunities and challenges in informing the development of interventions. Asia Pac J Oncol Nurs.

[18] (2018). The state of LTE. OpenSignal.

[19] (2015). Ericsson mobility report: 70 percent of world's population using smartphones by 2020. Ericsson.

[20] Dorsey ER, Yvonne Chan YF, McConnell MV, Shaw SY, Trister AD, Friend SH (2017). The use of smartphones for health research. Acad Med.

[21] Heynsbergh N, Heckel L, Botti M, Livingston PM (2018). Feasibility, useability and acceptability of technology-based interventions for informal cancer carers: a systematic review. BMC Cancer.

[22] Heynsbergh N, Heckel L, Botti M, Livingston PM (2019). A smartphone app to support carers of people living with cancer: a feasibility and usability study. JMIR Cancer.

[23] Sanmartin C, Murphy K, Choptain N, Conner-Spady B, McLaren L, Bohm E, Dunbar MJ, Sanmugasunderam S, De Coster C, McGurran J, Lorenzetti DL, Noseworthy T (2008). Appropriateness of healthcare interventions: concepts and scoping of the published literature. Int J Technol Assess Health Care.

[24] Heynsbergh N, Heckel L, Botti M, O SC, Livingston PM (2019). Development of a smartphone app for informal carers of people with cancer: processes and learnings. JMIR Form Res.

[25] Colorafi KJ, Evans B (2016). Qualitative descriptive methods in health science research. HERD.

[26] Cherryholmes CH (1992). Notes on pragmatism and scientific realism. Educ Res.

[27] Heynsbergh N, Botti M, Heckel L, Livingston PM (2018). Caring for the person with cancer: information and support needs and the role of technology. Psychooncology.

[28] Still B, Crane K (2017). Fundamentals of user-centered design: a practical approach.

[29] Srivastava A, Thomson SB (2009). Framework analysis: a qualitative methodology for applied policy research. J Adm Gov.

[30] Tong A, Sainsbury P, Craig J (2007). Consolidated criteria for reporting qualitative research (COREQ): a 32-item checklist for interviews and focus groups. Int J Qual Health Care.

[31] Bradshaw C, Atkinson S, Doody O (2017). Employing a qualitative description approach in health care research. Glob Qual Nurs Res.

[32] Treanor CJ, Santin O, Prue G, Coleman H, Cardwell CR, O'Halloran P, Donnelly M (2019). Psychosocial interventions for informal caregivers of people living with cancer. Cochrane Database Syst Rev.

[33] Manne SL, Kashy DA, Kissane D, Zaider T, Heckman CJ, Penedo FJ, Myers S (2019). Relationship intimacy processes during treatment for couple-focused interventions for prostate cancer patients and their spouses. J Psychosoc Oncol.

[34] Ullgren H, Tsitsi T, Papastavrou E, Charalambous A (2018). How family caregivers of cancer patients manage symptoms at home: a systematic review. Int J Nurs Stud.

[35] Bottorff JL, Oliffe JL, Halpin M, Phillips M, McLean G, Mroz L (2008). Women and prostate cancer support groups: the gender connect?. Soc Sci Med.

[36] Wang T, Molassiotis A, Chung BP, Tan JY (2018). Unmet care needs of advanced cancer patients and their informal caregivers: a systematic review. BMC Palliat Care.

[37] Carter N, Bryant-Lukosius D, DiCenso A, Blythe J, Neville AJ (2010). The supportive care needs of family members of men with advanced prostate cancer. Can Oncol Nurs J.

[38] Williams KC, Hicks EM, Chang N, Connor SE, Maliski SL (2014). Purposeful normalization when caring for husbands recovering from prostate cancer. Qual Health Res.

[39] Luo X, Gao L, Li J, Lin Y, Zhao J, Li Q (2020). A critical literature review of dyadic Web-based interventions to support cancer patients and their caregivers, and directions for future research. Psychooncology.

[40] Hensher M, Cooper P, Dona SW, Angeles MR, Nguyen D, Heynsbergh N, Chatterton ML, Peeters A (2021). Scoping review: development and assessment of evaluation frameworks of mobile health apps for recommendations to consumers. J Am Med Inform Assoc.

[41] Ugalde A, Winter N, Sansom-Daly UM, Rhee J, Jongebloed H, Bergin RJ, Livingston PM (2021). Effective integration of caregivers and families as part of the care team for people with cancer. Aust J Gen Pract.

